# Molecular Analysis of Xmn1-Polymorphic Site ´5 to G*γ* of the *β*-Globin Gene Cluster in a Saudi Population of Jazan Region in Correlation with Hb F Expression

**DOI:** 10.1155/2022/1717207

**Published:** 2022-03-21

**Authors:** Abozer Y. Elderdery, Abdullah Alsrhani, Badr Alzahrani, Muhammad Atif, Ahmed I. Refaiy, Hussain Shiwani, Amin Abbas, Dawelbiet A. Yahia

**Affiliations:** ^1^Department of Clinical Laboratory Sciences, College of Applied Medical Sciences, Jouf University, Sakaka, Saudi Arabia; ^2^Health Sciences Research Unit, Jouf University, Sakaka, Saudi Arabia; ^3^Laboratories and Blood Banks Administration, Jazan, Saudi Arabia; ^4^King Fahad Central Hospital, Jazan, Saudi Arabia; ^5^Alreqqa Medical Center, Madinah, Saudi Arabia; ^6^Department of Biochemistry, Faculty of Medicine, University of El Imam El Mahdi, Kosti, Sudan

## Abstract

The southern part of Saudi Arabia has an ethnically diverse population where sickle-cell anemia (sickle cell disease) is common, but little is known about its *β*s haplotypes. The goal of the current study is to ascertain the prevalence of the Hb S gene with analysis of Xmn1 ′5 to G*γ* haplotype among the Saudi population in the Jazan area. Initially recorded findings of (1) Hb S gene and (2) hematological parameters with Hb F levels were collected from 5990 participants. Then, the second series of 70 different patients with established sickling disease and 30 healthy individuals as a control group was recruited, in which the genotype of Xmn1 ′5 to G*γ*-SNP was performed by PCR-RFLP. In the first series, the prevalence of Hb types was AA at 86.8% (*N* = 5198), AS at 12.4% (*N* = 745), and SS at 0.8% (*N* = 47). Of the second series, three patients (4.3%) were (±) Xmn1 ′5 to G*γ* and 67 (95.7%) were (−/−) in Xmn1 ′5 to G*γ*. In the controls, the (±) Xmn1 ′5 to G*γ* was observed in only one individual (3.3%), aged 30. These findings possibly represent a new Saudi haplotype, [±] Xmn1 ′5 to G*γ*. Our results demonstrate that most patients with SCD in Jazan have [−/−] Xmn1 with higher levels of Hb F and positive Xmn1 ′5 to G*γ* normally associated with a low level of Hb F.

## 1. Introduction

Hemoglobin (Hb) S is the type of qualitative Hb abnormalities that have been reported as common in southern and western parts of Saudi Arabia [1, 2]. A study found that Hb S has a much higher incidence in Saudi Arabia (2–27%) [3] compared with some areas of the Arabian Peninsula [4]and Jordan (0.44%) [5]. On the other hand, Hb AS frequency is high in Bahrain states (11–18%) (11–18%) [6] and Kuwait (6%) [7]. Sickle cell anemia (SCA) is a particularly common genetic disorder in some parts of Saudi Arabia [2, 8]. The polymorphism in the *β* chain of Hb S and patient prognosis can be predicted by combining the levels of Hb F with a determination of which Xmn1 ′5 to G*γ* is present [9]. High levels of the former can interact with the latter to reduce disease severity [10]. Five different *β*s haplotypes are named after the geographical area where they prevail [7]. They are designated as Senegal, Benin, Bantu, Cameroon, and Arab-Indian haplotypes. The latter was discovered with the presence of Xmn1 ′5 to G*γ* in the Arabian Gulf region and India with fewer clinical symptoms as the Hb F is present in high levels in comparison with the haplotypes in Africa [8, 9]. The Senegal and Arab-Indian haplotypes are positive in Xmn1 ′5 to G*γ*, having the same mutation (C--T) and also a high level of Hb F as well [8, 10]. These haplotypes are used as genetic markers for the phenotypic heterogeneity of patients with SCA.

Multiple techniques are used for Hb S detection, including blood morphology, special tests (sickling and solubility), electrophoresis (alkaline and acid), isoelectric focusing, cation exchange high performance liquid chromatography (CE-HPLC), and molecular genetics [11]. This latter is a foundational technique commonly used in DNA analysis for *β*s haplotype identification, detecting the type of mutation. It included restriction fragment length polymorphism (RFLP), allele specific oligonucleotide (ASO), and amplification refractory mutation system (ARMS), LAMP assay and DNA sequencing [12, 13]. RFLP was used in this study for detecting *β*s haplotypes among Saudi patients with the S gene in the Jazan area.

SCA is a type of Hb variant, most commonly detected in most African and Asian countries, including Saudi Arabia, where it is a special problem in many areas [3, 5]. There is a polymorphism in the *β*-globin gene of Hb S, and patient prognosis can be predicted by combining the Hb F levels with a determination of which *β*s haplotypes are present [9, 10]. High levels of the former can act with the latter to alleviate disease severity and in turn lower mortality rate [10, 13, 14]. Approximately 95% of *β*^s^ haplotypes are denoted “typical” and the remainder atypical *β*^s^ haplotypes [15]. The former group includes the five types mentioned above, while the latter rarely occurs and may originate from a Benin or Bantu chromosome by only one mutation at the 5′ end [14]. All these forms are responsible for SCA with varying degrees of clinical symptom [14–18]. Thus, the objective of the current research is to ascertain the prevalence of the S gene by analysis of the Xmn1 ′5 to G*γ* haplotype in a Saudi population from the Jazan area.

The study population in the second series was performed on 100 participants, involving 70 patients with SCD and 30 healthy individuals as controls. Participants were screened during a premarital program in King Fahad Central Hospital in the Jazan region, Saudi Arabia, from January to December 2018. The collected information was retained confidentially, and data with the potential to reveal participant identities was omitted. The ethical committee of Jouf University approved the research.

## 2. Research Investigations

The level of Hb F has not been influenced by age, as all participants of the study were young adults recruited from the premarital centre. Hb variants, namely, Hb S, Hb A, Hb F, and Hb A2, were detected quantitatively, using the chromatography technique. Hematology analyser performed blood profiles, namely, hemoglobin (Hb), packed cell volume (PCV), mean cell volume (MCV), mean cell Hb (MCH), mean cell Hb concentration (MCHC), and red blood cell (RBC).

The polymorphism of Xmn1 *γ*G globin tests (−158C > *T*) was performed using polymerase chain reaction-restriction fragment length polymorphism (PCR-RFLP) and with Xmn1 as a restriction enzyme. The process involved a buffy coat to extract genomic DNA, standardized primers, and a UV NanoDrop 2000. DNA purity was initially quantified for each specimen before further analysis. Primers used were 5-AAC-TGT-TGC-TTT-ATA-GGA-TTTT-3 and 5-AGG-AGCTTATTGATAACCTCAGAC-3, which amplified the region from 5 to the *γ*G gene, which is a 650-bp fragment as described by Moez et al. [9]. Amplified products were then subjected to Xmn1 restriction for fragment visualization. Fragments visualized were GAA NN↑NN TTC 3 and 3 CTT NN↑NN AAG5 and these were classified into three polymorphisms: the homozygous state (CC) as a wild-type allele, the heterozygous form (CT), and the homozygous state (TT).

## 3. Statistical Analysis

Findings were examined using the SPSS, Version 25 (https://www.ibm.com/products/spss-statistics). Genotype distribution between SCD patients and the controls was compared using the Fisher exact test to count data. The odds ratios (OR) for genotypes of Xmn1 ′5 to G*γ* frequency in all subjects were 95% confidence interval (CI). We considered *P* < 0.05 to be statistically significant.

## 4. Ethical Consideration

This research was approved by the Jouf University, Ethics Committee, No. 39/9-19-4.

## 5. Results

Haemogram indices in individuals with the S gene against the control group were as follows:

- Mean blood profiles for healthy individuals with normal parameters [5198 (86.8%)] were Hb 15.8 g/dL, PCV 46.6%, MCV 83.3 fL, MCH 26.2 pg, MCHC 37.1 g/dL, and RBC 4.6 × 103/cmm.
- Mean blood profiles for participants with Hb SS and Hb AS were Hb 8.1 and 12.6 g/dL, PCV 26.7 and 37%, MCV 78.2 and 72.6 fL, MCH 25.6 and 26.7 pg, MCHC 35.6 and 36.2 g/dL, platelets 421 × 103 and 309 × 103/cmm, and RBC 3.2 × 106 and 3.8 × 106/cmm (Table 1). Our patient results were different compared to healthy individuals of Saudi Arabia [19].

Differential counts of WBC in the study population were as follows:

- Mean differential counts of WBC for healthy individuals with normal parameters [5198 (86.8%)] were WBC 5.6 × 103/cmm, neutrophil 47%, lymphocyte 33%, monocyte 8%, eosinophil 3.4%, and basophil 0.9%.
- Mean differential counts of WBC for participants with Hb SS and Hb AS were WBC 12.7 × 103 and 6.9 × 103/cmm, neutrophil 62 and 51%, lymphocyte 28 and 34%, monocyte 8.9 and 8.2%, eosinophil 2.7 and 3.2%, and basophil 0.8 and 0.9%, respectively (Table 2).

Platelet count among study participants: PLt counts in participants with Hb SS were significantly higher than in those with AA at 398 (125 × 10^9^/L) with *P* value 0.05, as opposed to 245 (95 × 10^9^/L) and AS, 289 (114 × 10^9^/L). PLt counts were also found to be significantly higher in the latter compared to participants with Hb AA (Table 1).

**Figure 1:**
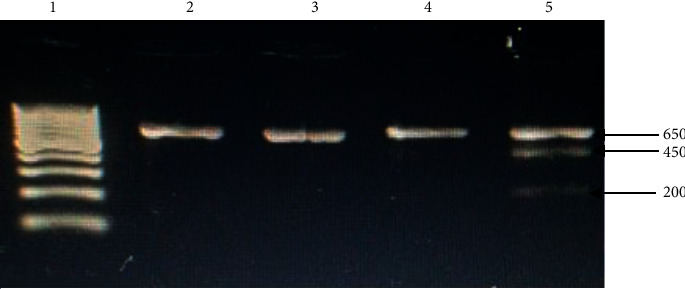
Some of RFLP products of Xmn1 ′5 to G*γ* of sickle patients and healthy individuals. Lane 1 demonstrates the DNA molecular weight marker with a hundred base-pair ladder. Lanes 1 to 4 and 8 are the DNA which is (−/−), whereas Lane 5 is the DNA which is (±) for Xmn1 ′5 to G*γ*.

**Figure 2:**
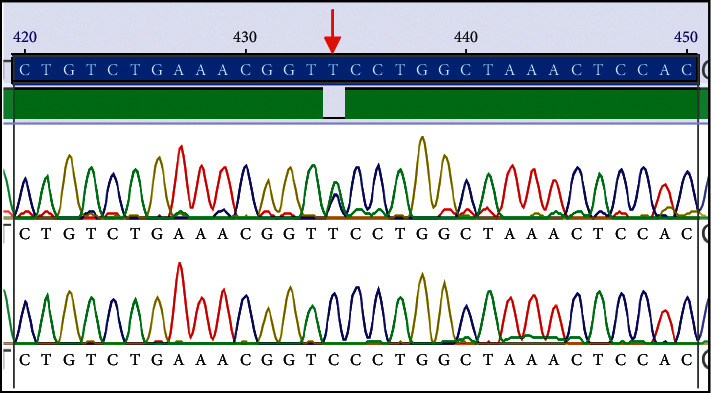
Chromatograms from XmnI reactions. Uppermost lane highlighted in blue is the consensus DNA sequence where the ′5 to G*γ*-SNP is indicated by the red arrow. Top: RFLP positive sample; bottom: RFLP negative sample.

*Xmn1 ′5 to Gγ results:* in the second series (Table 2), genotyping of the −158G*γ* (CvT) Xmn1 polymorphism revealed that, of the SS class, 95.7% were found to be a wild-type allele (specifically homozygous CC), and 4.3% were heterozygous (CT). The former was undigested with only one band (650 bp), and the latter partially digested (±) with three bands, 650, 450, and 200 bp (Figure 1). In controls, the ± Xmn1 ′5 to G*γ* was observed in only one individual of thirty (3.3%), with the remainder found to have a wild-type allele, homozygous state (CC), 96.7%, as shown in Figures 1 and 2.

**Figure 3:**
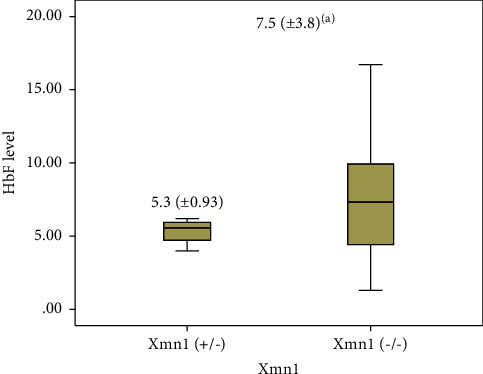
Demonstrations of platelet count among study participants. Key: significance determined by comparison of each group with control indices. (a) = *P* < 0.05; ^*∗*^statistical significance is shown in parenthesis.

Table 2 also demonstrates that the OR indicates that there is a strong negative correlation between CC and CT. Furthermore, the *P* value (*P*=1.0) suggests that there is no significant variation between controls and patients regarding CC and CT polymorphisms. 95% CI indicates that these values calculated are between 0.11 and 4.52. Patients and controls were taken from the same societal group, and judging from statistics, most participants in this group possess a wild allele (specifically homozygous CC), 95.7% in patients, and 96.7 in controls. These statistics are calculated, using the Fisher exact test, where the input is the 2 by 2 contingency table holding the percentages of patients and controls who possess CC and CT. TT is omitted from the calculation because the percentage of patients and controls who hold TT is zero. This mutant allele (TT) with two bands, 450 and 200 bp, might represent a new Saudi haplotype. Among SS and AA classes, cases with the (CT) heterozygous genotype of Xmn1 ′5 to G*γ* had lower levels of Hb F compared to those with the wild-type allele, (−/−) Xmn1 ′5 to G*γ* at 5.3% and 7.5%, respectively (*P* < 0.005) (Figure 3).

## 6. Discussion

Saudi Arabia is a large country comprising several tribes of differing ethnicity. Based on initial data from the Jazan area premarital centre, it can be suggested that such diversity will be associated with a raised frequency of sickle Hb. As often found in ethnic groups where the *β*s allele is frequent, this Arab diversity will also be associated with variations in Hb type [20, 21]. Therefore, as *β*s mutations vary according to DNA background, a study was undertaken by Elderdery et al. to evaluate the possibility of new haplotypes being present other than those already known [13].

Additionally, the study researched Hb F levels associated with Xmn1 ′5 to G*γ*, as an indicator of disease severity [10]. To address this objective, the current study was extended to characterize the genotype frequency of the −158G*γ* (C⟶T) Xmn1 polymorphism [13, 22, 23]. The S (sickle) gene is a monogenic disorder created by a single A/T mutation in the sixth position of the *β*-chain; however, other clinical phenotypes are involved. Xmn1 ′5 to G*γ* analysis for S gene presence was based on RFLP for published SNPs and it was detected within the genomic region stretching from the ´5 to G*γ* globin to *β*-globin gene [9]. Here, S gene frequency was found in only 4.3% of patients with the heterozygous (±) form and 95.7% of those who were homozygous (-/-).

This finding is in disagreement with existing studies of the Arab-Indian haplotype, which found a high frequency of the S gene in patients who are Xmn1 ′5 to G*γ* positive [24, 25]. Furthermore, our findings were also dissimilar to findings from SCA patients from Yemen [26] and Senegal [27]. Our hypothesis is in agreement with similar studies undertaken in Sudan (13) and Palestine [28], however. Here, Cameroon and Benin haplotypes predominate, respectively, with low levels of Hb F. It is also under a study from Tunisia which found that Xmn1 ′5 to G*γ* was largely (−/−) a wild genotype [29]. It is known that the Arab-Indian haplotype is widespread throughout the Middle East (including Saudi Arabia) [25, 30], but our findings indicate that this haplotype has only a limited presence among sickle patients in the Jazan area.

An atypical haplotype is one caused by recombination of common ones, meaning they are arranged into separated block-like structures, over the human genome [31]. The current study revealed a general absence of the S (sickle) gene among 94.9% of patients from Xmn1 ′5 to G*γ* (Figures 1 and 2). This absence is probably due to different genetic backgrounds within the Saudi population and would be following Elderdery et al. (Sudan) and Steinberg et al. in African Americans and widespread presence of the atypical *β*-globin gene [13, 32]. This may support the hypothesis that a new haplotype may be present in sickle-cell patients from specific Saudi tribes.

Both Senegal and Arab-Indian haplotypes are accompanied by a higher Hb F presence between Xmn1 ′5 and G*γ* [10, 27, 33], but sickle-cell patients of the Benin haplotype are accompanied with intermediate levels only [34]. Furthermore, sickle patients of Bantu or Central African Republic Haplotypes are documented with the lowest levels of Hb F [13]. Based on regional genetic epidemiological studies, it was detected that the predominant ethnicity of sickle patients with the typical *β*-s haplotype in Saudi Arabia is the Arab-Indian [25]. This current study however did not analyze *β*-globin gene haplotypes, which is a limiting factor. *β*s haplotypes are genetic factors indicating Hb F levels in sickled patients because it is known that higher levels have an impact in lowering the clinical severity of the disease [35]. Among both SS and AA classes, only 5.3% of cases with the (CT) heterozygous genotype (Xmn1 ′5 to G*γ*) had lower levels of Hb F compared with 7.5% of those with the wild-type allele, (−/−) Xmn1 ′5 to G*γ*, *P* < 0.05 (Figure 2).

This is unlike other findings among sickled patients from other areas of the same country but is similar to studies among other ethnic groups [13]. Within Saudi controls with Hb AA, 3.5–8.5% showed heterogeneity of Hb F expression, higher than that for such controls in other countries. However, this may result from difference in local genetic background, which may reveal variation in clinical features of SCD [32, 33]. In contrast, some studies have reported that SCA patients in the Gulf region and Asian countries including India had fewer clinical symptoms. This would be because Hb F is found there at a higher level than in African countries, owing to the variation in *β*s haplotypes [33–35].

## 7. Conclusion

A study of polymorphisms of the Xmn1 *γ*G globin (−158C > *T*) from subjects in Saudi Arabia's Jazan area would identify specific genetic backgrounds common to the Saudi community as a whole, thus this region should be considered for further future research. This study reveals that most cases of SCA in Jazan have [−/−] Xmn1 having higher levels of Hb F and positive Xmn1 ′5 to G*γ*, which is normally associated with a significantly low level of Hb F compared with negative Xmn1 ′5 to G*γ*. Additionally, tested patients and controls were not homozygous (TT) for the mutant allele, reflecting, and explaining the limited effect of polymorphism in raising Hb F levels. Focusing on Jazan area as the sickle Hb is more prevalent over there and therefore, further research from other areas of Saudi Arabia and with larger sample sizes and different age group above six months together with the molecular analysis of *β*S haplotypes, would better evaluate this probable association with SCD [36].

## Figures and Tables

**Table 1:** Complete haemogram indices in sickle patients against the control group.

Blood parameters	Control	Hb AS	Hb SS
RBC x 109/L (±SD)	4.6 × 106 (0.5)	3.8 × 106 (0.4)	3.2 × 106 (±0.4) (a)
Hb g/dl (±SD)	15.8 (2.2) (a)	12.6 (1.1) (a)	8.1 (1.2) (a)
MCH/pg (±SD)	26.2 (1.1) (a)	26.7 (1.5) (c)	25.6 (2.1) (c)
MCHC g/dl (±SD)	37.1 (1.1) (a)	36.2 (1.6) (c)	35.6 (1.8) (c)
PCV % (±SD)	46.6 (4.2) (a)	37.0 (5.6) (a)	26.7 (4.2) (a)
MCV fl (±SD)	83.3 (5.3)	72.6 (6.5) (a)	78.2 (7.9) (a)
RDW fl (±SD)	42.1 (2.6)	43.7 (3.5)	83.8 (4.3) (a)
WBC x 109/L (±SD)	5.6 (1.7)	6.9 (1.3)	12.7 (3.2) (a)
Neut% (±SD)	47 (11)	51 (15)	62 (11) (a)
Lymph% (±SD)	33 (9)	34 (7)	28 (9)
Mono% (±SD)	8 (3.7)	8.2 (2.4)	8.9 (2.3)
Eosino% (±SD)	3.4 (1.9)	3.2 (2.1)	2.7 (2.8)
Baso% (±SD)	0.9 (1.2)	0.9 (0.7)	0.8 (0.5)
PLt x 10^9^/L (±SD)	245 (95)	289 (114) (a)	398 (125) (a)

Key: significance determined by comparison of each group with control indices. (a) = *P* < 0.05; ^*∗*^Statistical significance is shown in parenthesis.

**Table 2:** Distribution of genotype of the Xmn1 ′5 to G*γ* polymorphism in sickle patients and control subjects.

Genotype (−158C > *T*)	Patients N (%)	Controls N (%)	OR	95% CI	*P* value
CC (−/−)	67 (95.7)	29 (96.7)	0.743	0.11–4.52	1
CT (±)	3 (4.3)	1 (3.3)			
TT (+/+)	0 (0)	0 (0)	0	0	0

## Data Availability

The data used to support the findings of this study are included in the article. Should further data or information be required, these are available from the corresponding author upon request.

## Abbreviations

Hb: Hemoglobin
SCA: Sickle cell anemia
CE-HPLC: High performance liquid chromatography
ASO: Allele specific oligonucleotide
ARMS: Amplification refractory mutation system
PCV: Packed cell volume
MCV: Mean cell volume
MCH: Mean cell Hb
MCHC: mean cell Hb concentration
RBC: Red blood cell
RDW: Red cell distribution width
WBC: White blood cells
Neut: Neutrophil
Mono: Monocyte
Lymph: Lymphocyte
Eosino: Eosinophil
Baso: Basophil
PLt: Platelet
PCR-RFLP: Polymerase chain reaction-restriction fragment length polymorphism
SD: Standard deviation
OR: Odds ratio
CI: Confidence interval.
